# Supplementary material for: Dietary Polyphenol Intake and Gastric Cancer: A Systematic Review and Meta-Analysis
Source: Cancers (Basel). 2022 Nov 29;14(23):5878. doi: 10.3390/cancers14235878 (PMC9737802; doi:10.3390/cancers14235878)
Supplement: Supplementary file 1 [file cancers-14-05878-s001.zip › cancers-1941699-supplementary.pdf]

Supplementary materials

Table S1 - Full search terms for each database.

DATABASE	WEBSITE	SEARCH STRATEGY	TOTAL
Medline/ PUBMED via National Library of Medicine	http://www.ncbi.nlm.nih.gov/pubmed	(polyphenol OR provinols OR phenol OR flavonoid OR flavonol OR quercetin OR kaempferol OR myricetin OR isorhamnetin OR flavone OR luteolin OR apigenin OR flavanone OR eriodictyol OR hesperidin OR naringenin OR flavan-3-ol OR catechin OR epicatechin OR theaflavin OR anthocyanidin OR cyanidin OR delphinidin OR malvidin OR peonidin OR petunidin OR proanthocyanidins OR isoflavone OR anthocyanins OR benzoflavones OR beta-naphthoflavone OR biflavonoids OR chalcones OR diosmin OR rutin OR coumestrol OR genistein OR pterocarpum OR rotenone OR phloretin OR "polyphloretin phosphate" OR "phenolic acids" OR "hydroxycinnamic acids" OR "hydroxybenzoic acids" OR "hydroxyphenylacetic acids" OR lignans OR stilbenes OR alkylphenols OR alkylmethoxyphenols) AND ("Gastric Adenocarcinoma" OR "Neoplasm, Stomach" OR "Stomach Neoplasm" OR "Neoplasms, Stomach" OR "Gastric Neoplasms" OR "Gastric Neoplasm" OR "Neoplasm, Gastric" OR "Neoplasms, Gastric" OR "Cancer of Stomach" OR "Stomach Cancers" OR "Gastric Cancer" OR "Cancer, Gastric" OR "Cancers, Gastric" OR "Gastric Cancers" OR "Stomach Cancer" OR "Cancer, Stomach" OR "Cancers, Stomach" OR "Cancer of the Stomach")	1184
Embase	www.embase.com	('polyphenol'/exp OR polyphenol OR 'polyphenols'/exp OR polyphenols OR 'provinols'/exp OR provinols OR 'flavonoid'/exp OR flavonoid OR 'flavonoids'/exp OR flavonoids OR 'phenolic compounds'/exp OR 'phenolic compounds' OR (phenolic AND compounds) OR 'lignans'/exp OR lignans OR 'stilbenes'/exp OR stilbenes) AND ('stomach tumor'/exp OR 'stomach tumor')	1244
Scopus	www.scopus.com	(TITLE-ABS-KEY (polyphenol) OR TITLE-ABS-KEY (flavonoids) OR TITLE-ABS-KEY ("phenolic acids") OR TITLE-ABS-KEY (lignans) OR TITLE-ABS-KEY (stilbenes) AND TITLE-ABS-KEY ("gastric adenocarcinoma") OR TITLE-ABS-KEY ("stomach neoplasms"))	304
Lilacs	https://lilacs.bvsalud.org/	('polyphenol'/exp OR polyphenol OR 'polyphenols'/exp OR polyphenols OR 'provinols'/exp OR provinols OR 'flavonoid'/exp OR flavonoid OR 'flavonoids'/exp OR flavonoids OR 'phenolic compounds'/exp OR 'phenolic compounds' OR (phenolic AND compounds) OR 'lignans'/exp OR lignans OR 'stilbenes'/exp OR stilbenes) AND ('stomach tumor'/exp OR 'stomach tumor')	0
Web of science	www.webofknowledge.com	TS=((polyphenol OR provinols OR phenol OR flavonoid OR flavonol OR quercetin OR kaempferol OR myricetin OR isorhamnetin OR flavone OR luteolin OR apigenin OR flavanone OR eriodictyol OR hesperidin OR naringenin OR flavan-3-ol OR catechin OR epicatechin OR theaflavin OR anthocyanidin OR cyanidin OR delphinidin OR malvidin OR peonidin OR petunidin OR proanthocyanidins OR isoflavone OR anthocyanins OR benzoflavones OR beta-naphthoflavone OR biflavonoids OR chalcones OR diosmin OR rutin OR coumestrol OR genistein OR pterocarpum OR rotenone OR phloretin OR "polyphloretin phosphate" OR "phenolic acids" OR "hydroxycinnamic acids" OR "hydroxybenzoic acids" OR "hydroxyphenylacetic acids" OR lignans OR stilbenes OR alkylphenols OR alkylmethoxyphenols) AND ("Gastric	18

		Adenocarcinoma" OR "Neoplasm, Stomach" OR "Stomach Neoplasm" OR "Neoplasms, Stomach" OR "Gastric Neoplasms" OR "Gastric Neoplasm" OR "Neoplasm, Gastric" OR "Neoplasms, Gastric" OR "Cancer of Stomach" OR "Stomach Cancers" OR "Gastric Cancer" OR "Cancer, Gastric" OR "Cancers, Gastric" OR "Gastric Cancers" OR "Stomach Cancer" OR "Cancer, Stomach" OR "Cancers, Stomach" OR "Cancer of the Stomach"))	
OpenGrey	http://www.opengrey.eu	((polyphenol OR provinols OR phenol OR flavonoid OR flavonol OR quercetin OR kaempferol OR myricetin OR isorhamnetin OR flavone OR luteolin OR apigenin OR flavanone OR eriodictyol OR hesperidin OR naringenin OR flavan-3-ol OR catechin OR epicatechin OR theaflavin OR anthocyanidin OR cyanidin OR delphinidin OR malvidin OR peonidin OR petunidin OR proanthocyanidins OR isoflavone OR anthocyanins OR benzoflavones OR beta-naphthoflavone OR biflavonoids OR chalcones OR diosmin OR rutin OR coumestrol OR genistein OR pterocarpum OR rotenone OR phloretin OR "polyphloretin phosphate" OR "phenolic acids" OR "hydroxycinnamic acids" OR "hydroxybenzoic acids" OR "hydroxyphenylacetic acids" OR lignans OR stilbenes OR alkylphenols OR alkylmethoxyphenols) AND ("Gastric Adenocarcinoma" OR "Neoplasm, Stomach" OR "Stomach Neoplasm" OR "Neoplasms, Stomach" OR "Gastric Neoplasms" OR "Gastric Neoplasm" OR "Neoplasm, Gastric" OR "Neoplasms, Gastric" OR "Cancer of Stomach" OR "Stomach Cancers" OR "Gastric Cancer" OR "Cancer, Gastric" OR "Cancers, Gastric" OR "Gastric Cancers" OR "Stomach Cancer" OR "Cancer, Stomach" OR "Cancers, Stomach" OR "Cancer of the Stomach"))	2

Table S2 - Risk of bias assessments according to Newcastle-Ottawa Scale.

Reference	Study design	Selection	Comparability	Exposure/Outcome	Total
Garcia-Closas et al. [8]	Case-Control	***	**	*	**6**
Hirvonen et al. [12]	Prospective Cohort	****	**	***	**9**
Knekt et al. [16]	Prospective Cohort	****	**	***	**9**
Lagiou et al. [9]	Case-Control	***	**	*	**6**
Hernández-Ramírez et al. [25]	Case-Control	****	**	*	**7**
Ekström et al. [26]	Case-Control	****	**	**	**8**
Rossi et al. [14]	Case-Control	***	**	*	**6**
Hara et al. [10]	Prospective Cohort	****	**	***	**9**
Zamora-Ros et al. [27]	Prospective Cohort	***	**	***	**8**
Lin et al. [28]	Prospective Cohort	****	**	***	**9**
Woo et al. [13]	Case-Control	***	**	*	**6**
Petrick et al. [4]	Multicentric Case-Control	****	**	*	**7**
Wada et al. [29]	Prospective Cohort	****	**	***	**9**
Sun et al. [11]	Prospective Cohort	***	**	***	**8**
Yang et al. [30]	Case-Control	****	**	*	**7**
Vitelli-Storelli et al. [31]	Multicentric Case-Control	***	**	*	**6**
Vitelli-Storelli et al. [5]	10 Case-Control Studies	****	**	*	**7**
Rubín-García et al. [15]	Multicentric Case-Control	***	**	*	**6**
Kim et al. [32]	Case-Control	****	**	*	**7**

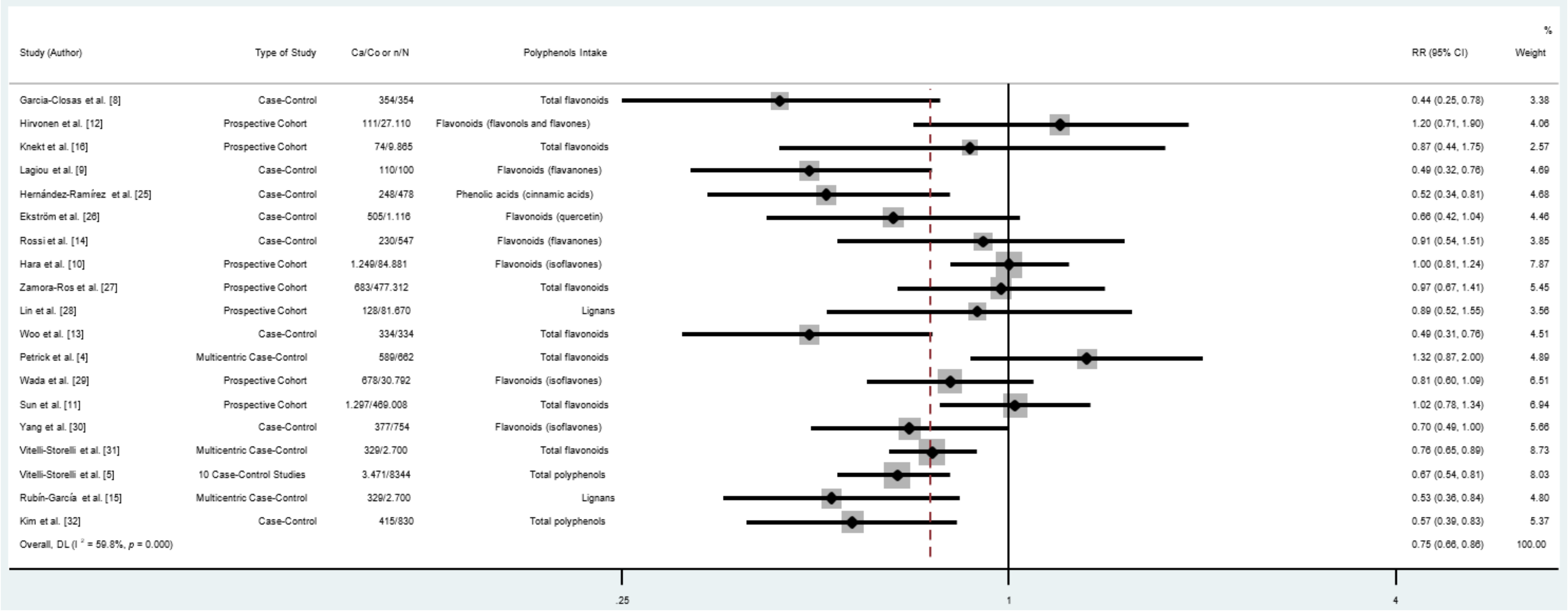

Figure S1 – Forest plot of polyphenol consumption and gastric cancer risk with other data combinations, considering the male sex and the cardia anatomical type as the first choice for studies whose results were separated according to these variables. Case–control and cohort studies are presented in chronological order of publication. Square box-es represent study-specific estimates. Markers vary in size according to weight assigned to each study, the size of each box reflects the study’s weight in the analysis, and horizontal lines represent 95% confidence intervals. Diamonds show the pooled effect. Abbreviations: RR, relative risk; Ca, num-ber of cases; Co, number of controls; n, number of events; N, number of patients.

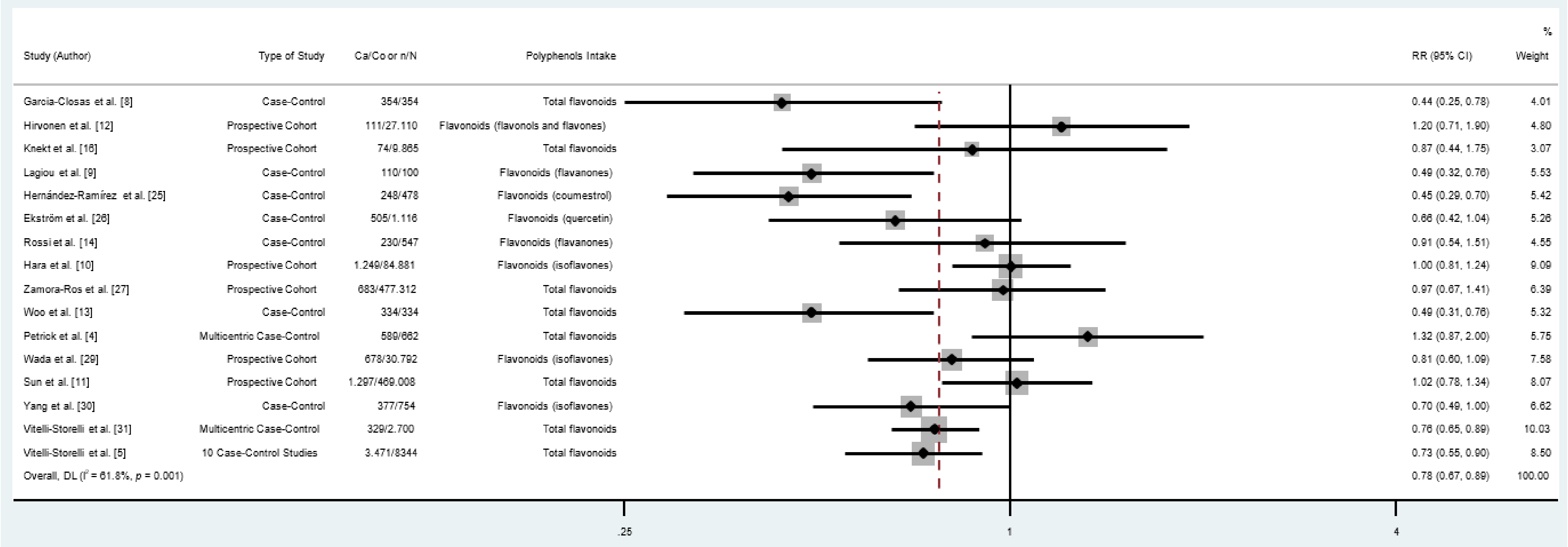

Figure S2 - Forest plot of flavonoid consumption and gastric cancer risk with other data combinations, considering the male sex and the cardia anatomical type as the first choice for studies whose results were separated according to these variables. Case–control and cohort studies are presented in chronological order of publication. Square boxes represent study-specific estimates. Markers vary in size according to weight assigned to each study, size of each box reflects the study's weight in the analysis, and horizontal lines represent 95% confidence intervals. Diamonds show the pooled effect. Abbreviations: RR, relative risk; Ca, number of cases; Co, number of controls; n, number of events; N, number of patients.

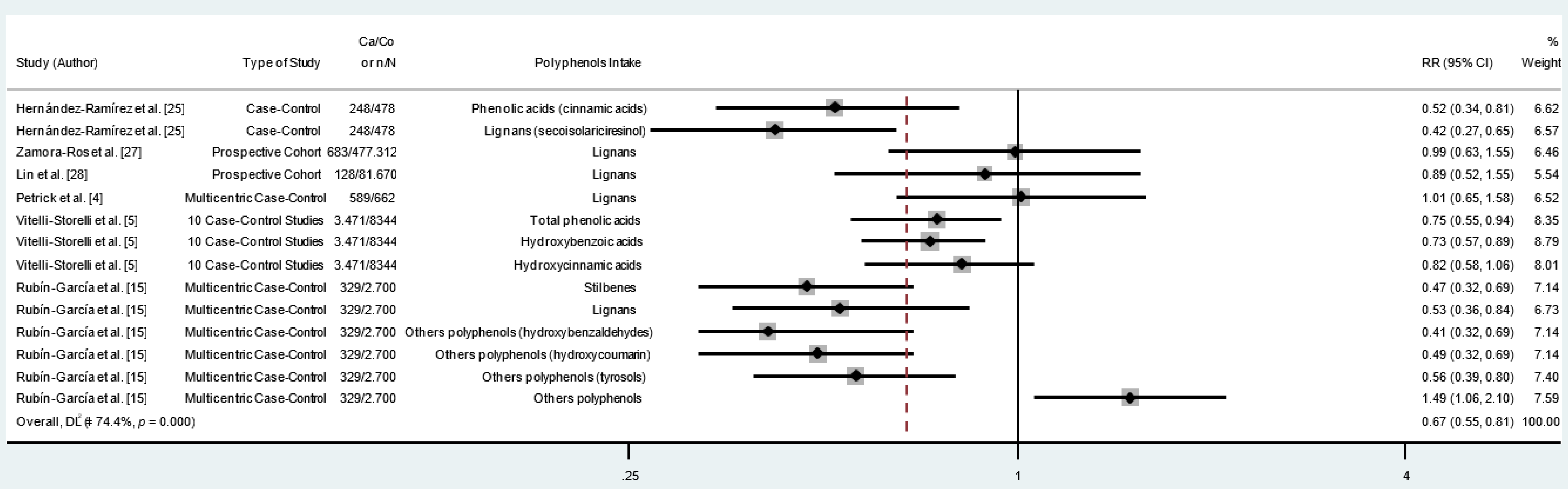

Figure S3 - Forest plot of consumption of lignans, phenolic acids, stilbenes, and other polyphenols and gastric cancer risk, with other data combinations, considering the male sex and the cardia anatomical type as the first choice for studies whose results were separated according to these variables. Case–control and cohort studies are presented in chronological order of publication. Square boxes represent study-specific estimates. Markers vary in size according to weight assigned to each study, size of each box reflects the study's weight in the analysis, and horizontal lines represent 95% confidence intervals. Diamonds show the pooled effect. Abbreviations: RR, relative risk; Ca, number of cases; Co, number of controls; n, number of events; N, number of patients.

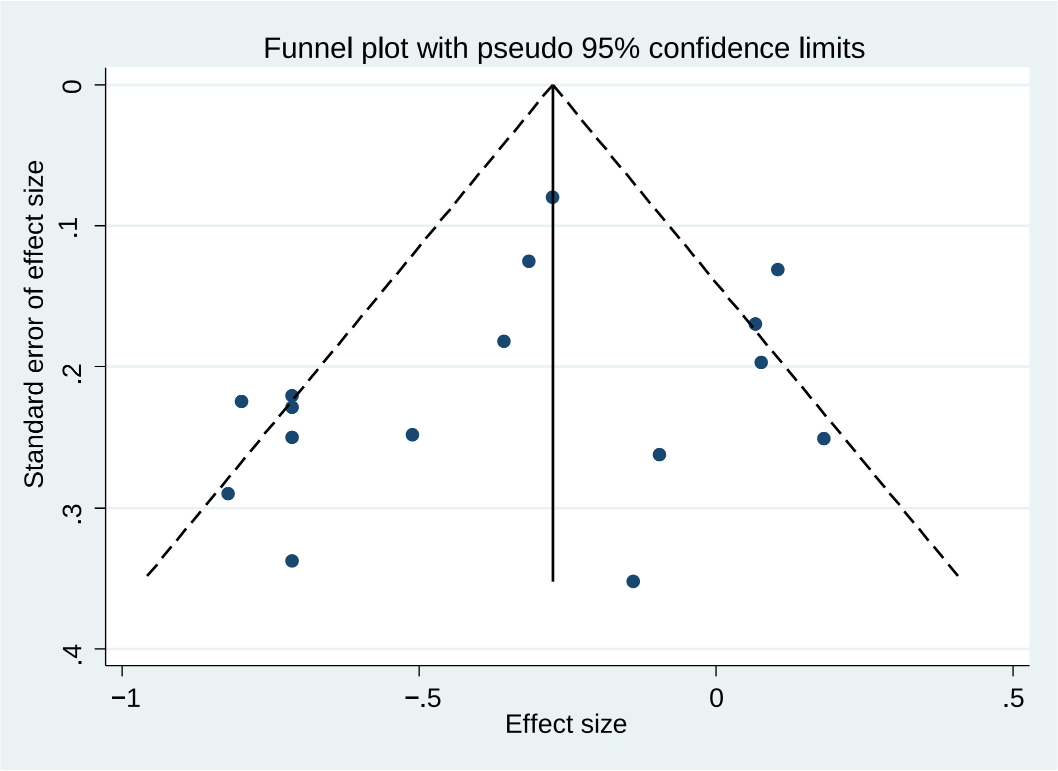

Figure S4 - Funnel plot of dietary flavonoid intake and gastric cancer risk.

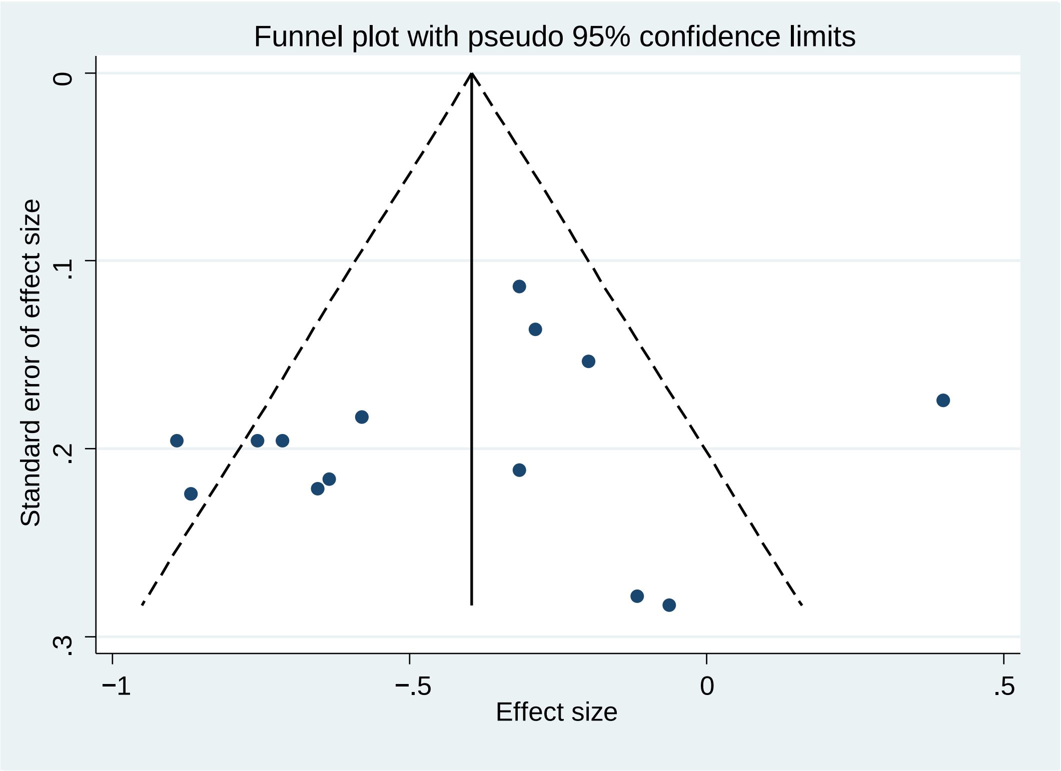

Figure S5 - Funnel plot of dietary lignans, phenolic acids, stilbenes, and other polyphenols and gastric cancer risk.
